# God's Ministers

**Published:** 1890-09-27

**Authors:** 


### Words of Consolation.

### GOD'S MINISTERS.

Just now the words Michaelmas and Michaelmas Day are familiar in our ears, but they recall no more to the mind of the many than that it is quarter day, and the rent is due. They do not know nor care to ask why it is so called. It is not altogether useless then to say that the 29th of September has been set apart by the Church, to the Glory of God and the Honour of Michael the Archangel and all the Hosts of Heaven. It will be interesting to find out what the Bible says about these wonderful beings, and see what help and consolation we may gain from it.

Most of us believe in a blind sort of way that there are angels, but who, or what they are, or why they are, we are quite ignorant of. Some people go so far as to imagine that when good men or women, and especially good children die, their souls become angels. But this is a very wrong notion, as the angels are beings quite distinct from men. St. Paul says, God made man a little lower than the Angels to crown him, in the Person of Jesus Christ, with glory and honour; while the Psalmist tells us God has made His Angels Spirits. All, however, were created by God's Word, whether they be things in Heaven or things on earth. The Angels are God's servants and messengers; they stand round about His Throne praising and adoring Him, and burning with zeal to carry His messages and speeding to and fro between Heaven and earth on the errands He gives them. In old time, they fought against Satan under Michael the Archangel and conquered. Now they are strong to help the sick and dying, they bring messages of grace to them, and carry safe to Paradise the souls released from earthly bondage.

Through the whole of the Scriptures, both in the Old and the New Testaments, we read of the Angels ministering to men's wants or inflicting on them necessary punishment. We first see them driving Adam and Eve from Paradise for their disobedience. Jacob is comforted by seeing in a dream the angels of God ascending and descending a ladder, the foot of which was on the ground, while at the top the Lord stood above it. The angels saved righteous Lot when they destroyed the cities of the plain for their wickedness. Their guardian care was extended to Daniel in the den of lions, and the three children were delivered from Nebuchadnezzar's fury and from the burning fiery furnace, and the soul of the beggar Lazarus was carried by them into Abraham's bosom. Truly may we say that the angels of God are round about them that fear Him.

In the New Testament, we read that the Angel Gabriel brought the message of Christ's Incarnation to the Blessed Virgin, and a multitude of the Heavenly Host shouted for joy, and praised God when the Babe of Bethlehem, the King of Glory, was born. What an immense interest have they always taken in man, and now they long to know all the mysteries which accompany his salvation! To the perfect Man, Christ Jesus, they brought consolation; after His temptation they ministered to Him, and during His agony in the garden they strengthened Him; they set free His risen body from the tomb, and at the Ascension like a cloud received Him out of sight.

And what they did for Jesus they will do for us. Our Lord tells us of little children that "their angels do always behold the face of our Father which is in heaven." It is the pure in heart, the innocent, the meek and lowly, who see God and are always before Him. Let us try to imitate the love and the burning zeal to do God service which distinguishes the holy angels. Let us strive after humility and purity, so that doing God's work here below, we may, when we have fought a good fight and finished our course, join those loyal hearts and true who are round about the throne.

